# P191: Validation tool to improve SSI surveillance in Swiss hospitals

**DOI:** 10.1186/2047-2994-2-S1-P191

**Published:** 2013-06-20

**Authors:** M-C Eisenring, S Kuster, H Sax, N Troillet

**Affiliations:** 1 Service de Maladies Infectieuses, Hôpital du Valais, Sion, Switzerland

## Introduction

The Swissnoso surgical site infection (SSI) surveillance module started in 2009. It includes currently >130 Swiss hospitals. To ensure quality and reliability of the surveillance system and of the data it provides, a validation tool was developed and implemented since 2012.

## Objectives

To describe the first results obtained with this validation tool.

## Methods

The validation, based on on-site visits to the participating hospitals, follows 2 steps: 1st, a questionnaire evaluates on a scale from 0 (bad) to 50 (excellent) the structure and the process of the surveillance; 2nd, 15 randomly selected patients’ charts are retrospectively reviewed in details (10 charts selected without consideration of SSI, 5 charts selected among patients with SSI).

## Results

As of March 2013, 23 hospitals had been evaluated. Their mean score on the evaluation scale was 34.6 (extremes: 20.0 – 45.6). The main issues detected in the structure and process of surveillance were: understaffing (slight in 21.7%, important in 8.7%), lack of participation in the training sessions organized by Swissnoso (41% of physicians and 29.6% of study nurses did not participate), problems in case inclusions (partially inadequate in 26.1%, incorrect in 4.3%), difficulties in finding the required medical information (13.1%), lack of supervision by a trained physician (rarely done in 8.7%, only sometimes done in 13.1%). Among the 234 cases reviewed, follow-up at one month was not available in 5%. Among 221 cases selected without consideration of SSI, SSI was missed in 5 (false negatives=2.3%). Among 103 cases with SSI, 18 (17.5%) were misclassified regarding the type of SSI (superficial, deep or organ/space).

## Conclusion

On-site visits allowed determining some gaps in the method of surveillance system at each hospital with potential impact on the accuracy of their results, in particular on their detected SSI rates.

## Disclosure of interest

None declared

